# An Age-Period-Cohort Analysis of Stroke Mortality Attributable to Low Physical Activity in China and Japan: Data from the GBD Study 1990–2016

**DOI:** 10.1038/s41598-020-63307-x

**Published:** 2020-04-16

**Authors:** Jinhong Cao, Ehab S. Eshak, Keyang Liu, Jin Yang, Krisztina Gero, Zhiming Liu, Chuanhua Yu

**Affiliations:** 1 Department of Epidemiology and Biostatistics, School of Health Sciences, Wuhan University, Wuhan, China; 2 Department of Public Health, Community and Preventive Medicine, Faculty of Medicine, Minia University, Minia, Egypt; 3 Public Health, Department of Social Medicine, Osaka University Graduate School of Medicine, Osaka, Japan; 4 Department of Social and Behavioral Sciences, Harvard T.H. Chan School of Public Health, Boston, USA; 5 Wuchang Hospital of Wuhan, Wuhan, China

**Keywords:** Stroke, Epidemiology, Risk factors

## Abstract

Stroke is the first and fourth leading cause of death in China and Japan, respectively. Physical inactivity was suggested to be one of the most important risk factors for stroke mortality. Therefore, this study aimed to assess long-term trends in stroke mortality attributable to low physical activity (LPA) in China and Japan during the period 1990–2016. Mortality data were obtained from the Global Burden of Disease Study 2016 (GBD 2016) and were analyzed with an age-period-cohort method. The age-standardized mortality rates (ASMRs) showed declining trends for LPA-attributable stroke mortality. The overall net drift per year was −1.3% for Chinese men, −2.9% for Chinese women, −3.9% for Japanese men, and −5.6% for Japanese women. In both countries, the local drift values were below zero in all age groups. The longitudinal age curves of LPA-attributable stroke mortality were higher in men than in women in all age groups. The period and cohort rate ratios showed similar downward patterns for both sexes, with a faster decline for women than for men. However, the physically active population is still small in both countries. Therefore, policymakers should further promote physical activity as one of the most recommended effective strategies in stroke prevention.

## Introduction

Stroke is a significant public health problem worldwide^1^. Even though in the past two decades, between 1990 and 2010, stroke mortality showed declining trends, it is still the first leading cause of death in China^2^, and the fourth in Japan^3^. Also, while the stroke mortality rate per 100,000 population decreased from 27.9 to 18.8 in Japan, the corresponding rates in China were 110.7 and 80.2, respectively^4^. To close the health gap between the two countries, it is crucial to identify controllable risk factors that led to a more substantial decrease in stroke mortality in Japan compared to China and to plan more effective prevention strategies and health-care management.

Previous case-control and cohort studies showed an inverse association between physical activity (PA) and the risk of stroke morbidity or mortality^5–8^. The Physical Activity Guidelines Advisory Committee reported that PA was associated with a 25–30% reduction in stroke risk^9^. Also, a nationwide cohort study in Korea identified lack of moderate- to vigorous-intensity PA as the second most important risk factor for stroke after high blood pressure^10^.

However, while several studies examined stroke mortality attributable to low physical activity (LPA), according to our knowledge, none of them had investigated age-specific mortality rates or explored the mechanisms that can justify the observed lifelong trends. Accordingly, comparing China and Japan, this study assessed the temporal trends and the autonomous impacts of birth cohort, chronologic age and time period on LPA-attributable stroke mortality between 1990 and 2016, utilizing an age-period-cohort model (APC) of estimable functions which were then processed by Microsoft Excel 2016 and employing data from the 2016 Global Burden of Disease Study (GBD 2016).

## Methods

### The data source

We accessed the free repository of the GBD 2016 which is managed by the Institute for Health Metrics and Evaluation (IHME) and contains indices for several environmental, behavioral and other risk factors of death (n = 84). The death rates since the 1990s to 2016 for 264 mortality causes according to age, sex and region were also available^11,12^. Among these data, we used the stroke mortality data in Japan (initially provided by the Japanese Social Health Insurance System)^13,14^ and in China (provided mainly through the Chinese Center for Disease Control and Prevention and other sources). Case definition of death from stroke was based on the clinical criteria of the World Health Organization and the 9th and 10^th^ revisions of the International Statistical Classification of Diseases^15^. PA was estimated as the average weekly Metabolic Equivalent of Task (MET) in minutes per week (min/wk) spent at home, work, transportation, and recreation^16,17^.

### Statistical analyses

Reaching a specific mortality endpoint represents not only the population’s death risk of that cause but also its aggregated lifelong risk factors, which cannot be precisely assessed via routine statistical analyses^18,19^. Thus, with the use of an APC method, we tested such interactive factors related to LPA-attributable stroke mortality including the biological and societal impacts of population’s chronologic age^20^, the health and life circumstances related to calendar period such as health policies, preventive strategies, medical advances and methods to certify deaths, and the common hazards shared by individuals in each generation’s cohort^20–24^.

Via the APC model Web Tool (Biostatistics Branch, National Cancer Institute, Bethesda, MD. https://analysistools.nci.nih.gov/apc/)^25^, we were able to estimate the net and local drifts, the longitudinal age curves, and the rate ratio (RR) for cohort/period. A drift (log-linear trend) represents the average annual change of the LPA-attributable stroke mortality rate over time according to the two axes of birth cohort and calendar period, with the net drift indicating the total percent change per year; while the local ones represent these specific changes in each age group. With the control for period variabilities, the longitudinal age curves can show the reference cohort’s age-specific LPA-attributable stroke mortality rates, and with the control for chronologic age and nonlinear components of period/cohort, the cohort/period RRs indicate the relative risks of LPA-attributable stroke mortality in each cohort/period in comparison to the referent one.

The study of the GBD 2016 provided the data of death, location, sex, age (progressive 5-year age groups), year (consecutive 5-year periods), disease cause, risk factors, metric (number and rate of cases) and value (upper and lower). The calculation formulas are as follows:

$${\rm{Population}}=\frac{{\rm{Number}}\,{\rm{of}}\,{\rm{cases}}}{{\rm{Rate}}\,{\rm{of}}\,{\rm{cases}}}\times 100,000$$

$${\rm{ASMRs}}=\frac{\sum ({\rm{Age}}\,{\rm{composition}}\,{\rm{of}}\,{\rm{standard}}\,{\rm{group}}\,{\rm{population}}\times \,{\rm{Age}}-{\rm{specific}}\,{\rm{mortality}}\,}{{\rm{Age}}\,{\rm{composition}}\,{\rm{of}}\,{\rm{standard}}\,{\rm{population}}}$$

In the GBD data, LPA was defined as <8000 MET min/wk^16,17^. The LPA-attributable stroke mortality was measured based on the following four components: the number of stroke deaths, the exposure levels for LPA, the relative risk of stroke mortality due to LPA, and the counterfactual level of LPA exposure. Thus, the LPA-attributable stroke mortality rate in a given year, location, sex and age was a result of the multiplication of the number of deaths from stroke and the population attributable fraction (PAF) for stroke-LPA pair; where the PAF identifies what percentage of stroke mortality could be avoided in a certain year if the level of LPA prior to that given year could have been reduced to the untruthful theoretical minimum risk exposure level (PA ≥ 8000 MET min/wk)^11^.

The sex-specific LPA-attributable stroke mortality rates in China and Japan were age-standardized based on the GBD 2013 global age-standard population^13^. The age-specific LPA-attributable stroke mortality rates between 1990 and 2016 were ranked into consecutive 5-year periods starting from a group aged 25–29 years to end with a group aged 75–79 years. We excluded those below 25 years and over 79 years because LPA-attributable stroke mortality is rare below the age of 25 years, and the GBD data could not verify the exact age after the age of 80 years.

The software of the Web tool is an open source program based on R language. Its core operation code is detailed in APC Web Tool’s open source project in Github (https://github.com/CBIIT/nci-webtools-dceg-age-period-cohort).

A specific set of estimable functions was used to conduct the APC analysis in our research and allowed us to assess the problem of perfect collinearity between age, period and cohort^26^, and the reference was set to the median values of age groups, periods, and birth cohorts. If the number of groups is even, the reference group is defined as the lower order of the middle two groups^25,26^. The significance of estimated parameters was tested by the Wald chi-square tests, and those of the period/cohort RRs slopes were tested by the general linear models that evaluated the significance of interaction terms between sex and calendar year/birth cohort. Version 9.4 of the SAS (SAS Institute Inc, Cary, NC) statistical software was used to run all statistical tests that were 2-tailed with p-values <0.05 were regarded as significant.

### Ethical statement

This study used deidentified publicly available data from the Global Burden of Di sease Study 2016 repository. Thus, ethical approval and an ethical statement from an institutional review board or ethics committee was not required for secondary analysis of data for China and Japan.

## Results

**Figure 1:**
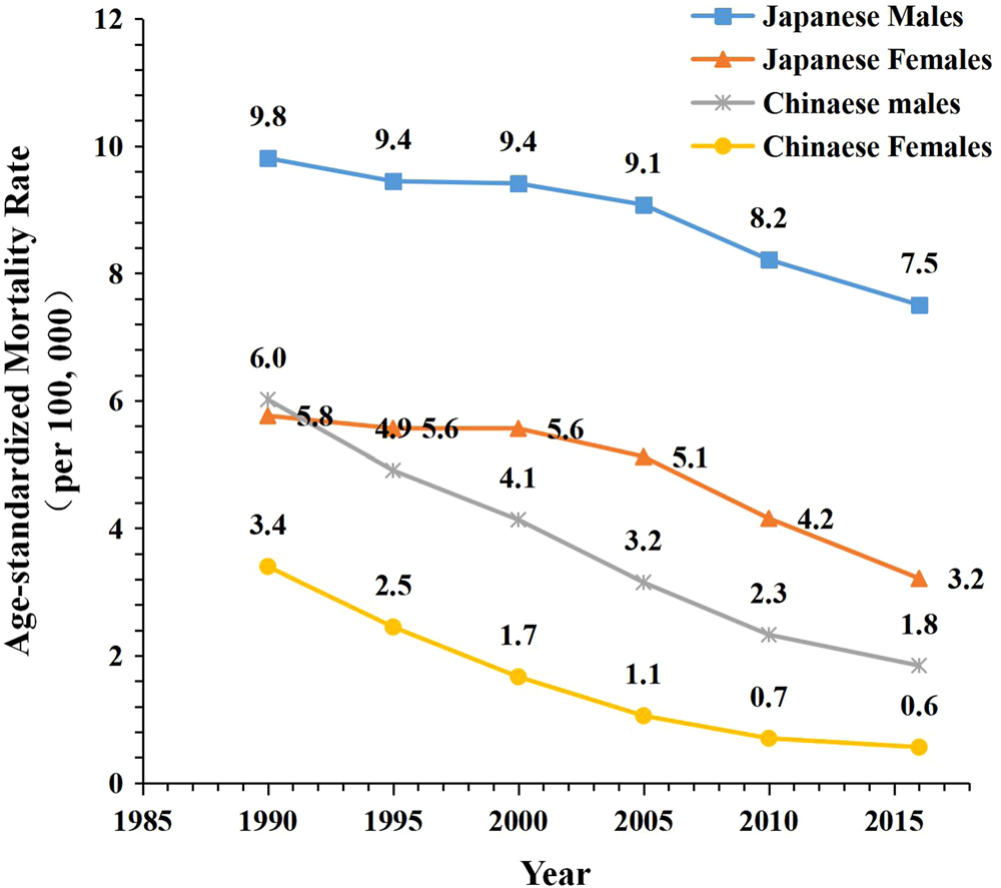
Trends of the age-standardized mortality rates (ASMR) per 100 000 population for LPA-attributable stroke mortality by sex in China and Japan, 1990 to 2016. Standardized to the GBD 2013 (Global Burden of Disease Study 2013) global age-standard population.

Figure 1 represents sex-specific ASMR trends for LPA-attributable stroke. Between 1990 and 2016, in China, the LPA-attributable stroke mortality rate per 100,000 population decreased from 9.8 to 7.5 for men and from 5.8 to 3.2 for women. The corresponding rates in Japan were 6.0 and 1.8 for men, and 3.4 and 0.6 for women, respectively. However, despite the overall declining trends, in China ASMRs were steady during the period between 1995 and 2000; the LPA-attribuλ stroke mortality rates per 100,000 population were 9.4 for Chinese men and 5.6 for Chinese women.

**Figure 2:**
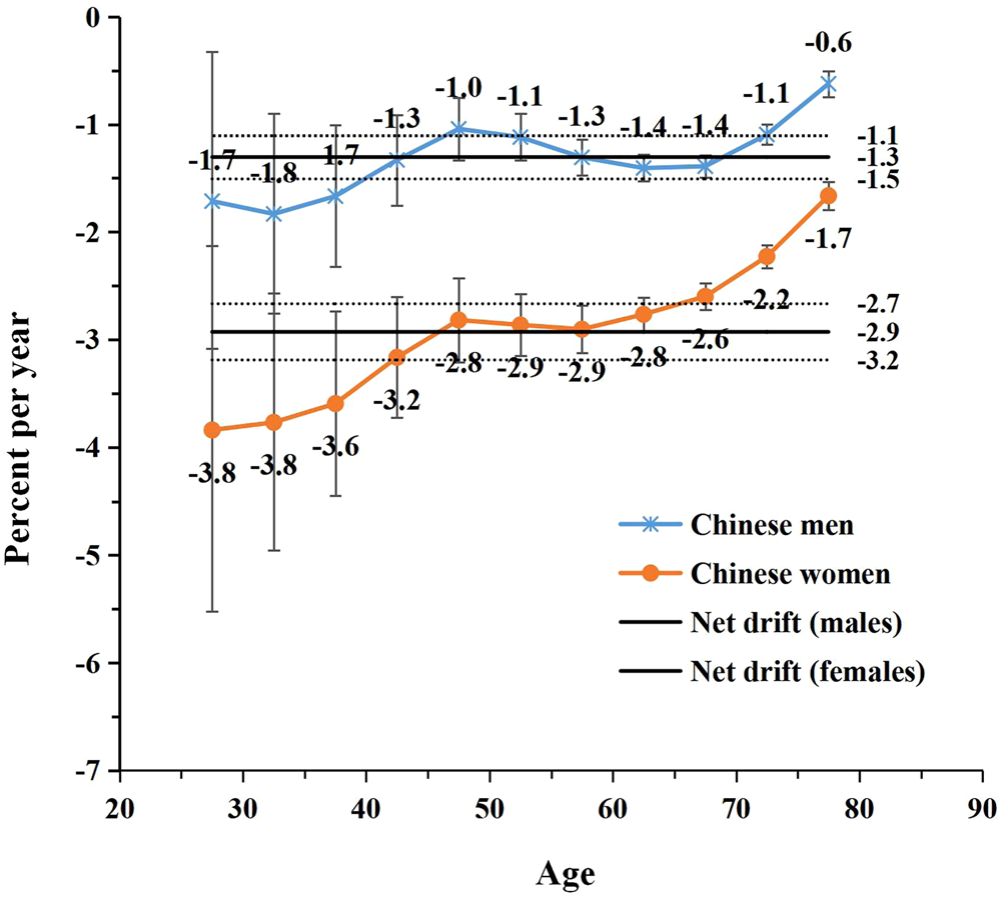
Local drift with net drift values for LPA-attributable stroke mortality in China. Age group-specific annual percent change (local drift) with the overall annual percent change (net drift) in high sodium-attributable stroke mortality rate. Net drift values are depicted as solid lines with dashed lines representing their 95% CIs. Error bars represent the 95% CIs for the local drift values.

**Figure 3:**
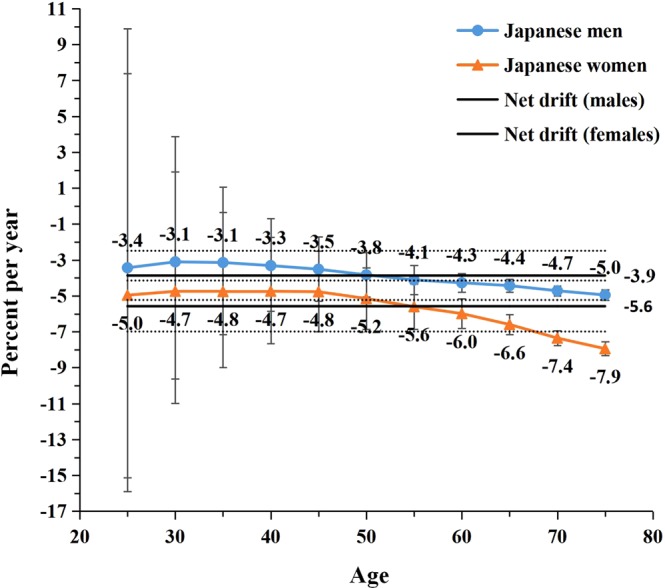
Local drift with net drift values for LPA-attributable stroke mortality in Japan. Age group-specific annual percent change (local drift) with the overall annual percent change (net drift) in high-sodium-intake-attributable stroke mortality rate. Net drift values are depicted as solid lines with dashed lines representing their 95% CIs. Error bars represent the 95% CIs for the local drift values.

The net drift – representing the annual percentage change of the expected overall age-adjusted rates – and the local drift – reflecting the expected age-specific rates over time – for LPA-attributable stroke mortality are shown in Figs. 2 and 3. During the period between 1990 and 2016, the overall net drift per year was −1.3% for Chinese men, −2.9% for Chinese women, −3.9% for Japanese men, and −5.6% for Japanese women (p < 0.01 for all). In both China and Japan, the local drift values were below zero in all age groups and were lower among women compared to men. However, the sex-specific local drift curves for LPA-attributable stroke mortality showed decreasing trends through increasing age groups in Japan, while the trend was opposite – increasing – in China (except for the age group of 45–55 years).

**Figure 4:**
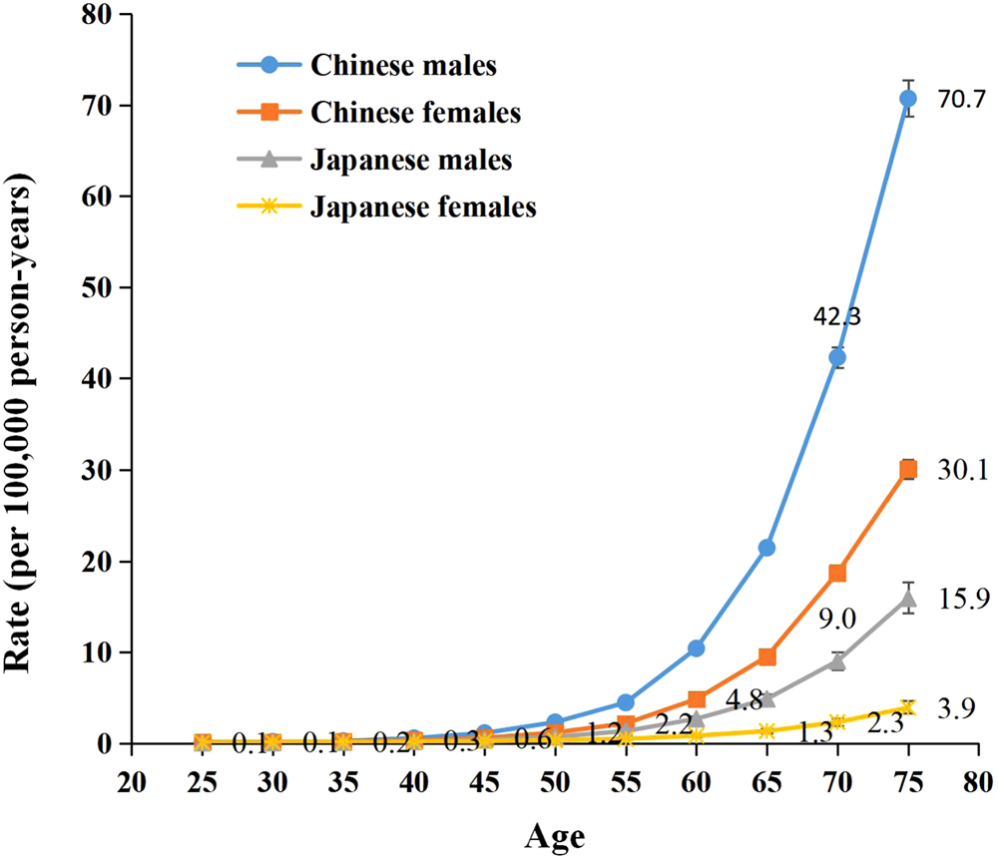
Longitudinal age curves of LPA-attributable stroke mortality in China and Japan. Fitted longitudinal age-specific rates of high-sodium-intake-attributable stroke mortality (per 100 000 person-years). Error bars represent the 95%CIs for the Longitudinal age curve values.

Figure 4 illustrates the longitudinal age curves of sex-specific LPA-attributable stroke mortality in China and Japan. In the same birth cohort, LPA-attributable stroke risk increased rapidly from the age of 55–59 years to peak at the age of 75–79 years for both sexes in both countries. The peak mortality rate per 100,000 person-years was 70.7 for Chinese men, 30.1 for Chinese women, 15.9 for Japanese men, and 3.9 for Japanese women. The LPA-attributable stroke risk was higher in men than in women in all age groups in both countries.

**Figure 5:**
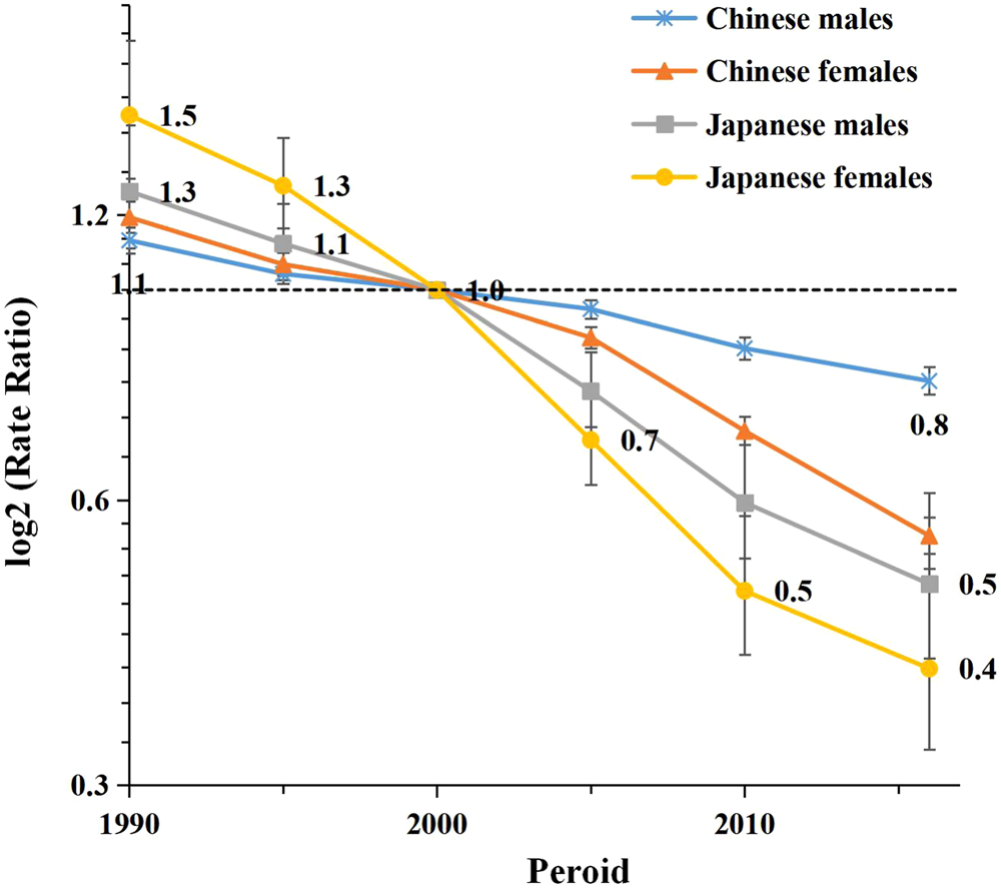
Period relative risks (RRs) of LPA-attributable stroke mortality rate by sex in China and Japan. The relative risk of each period compared with the reference one (year 2000) adjusted for age and nonlinear cohort effects. Error bars represent the 95%CIs for the period relative risks.

**Figure 6:**
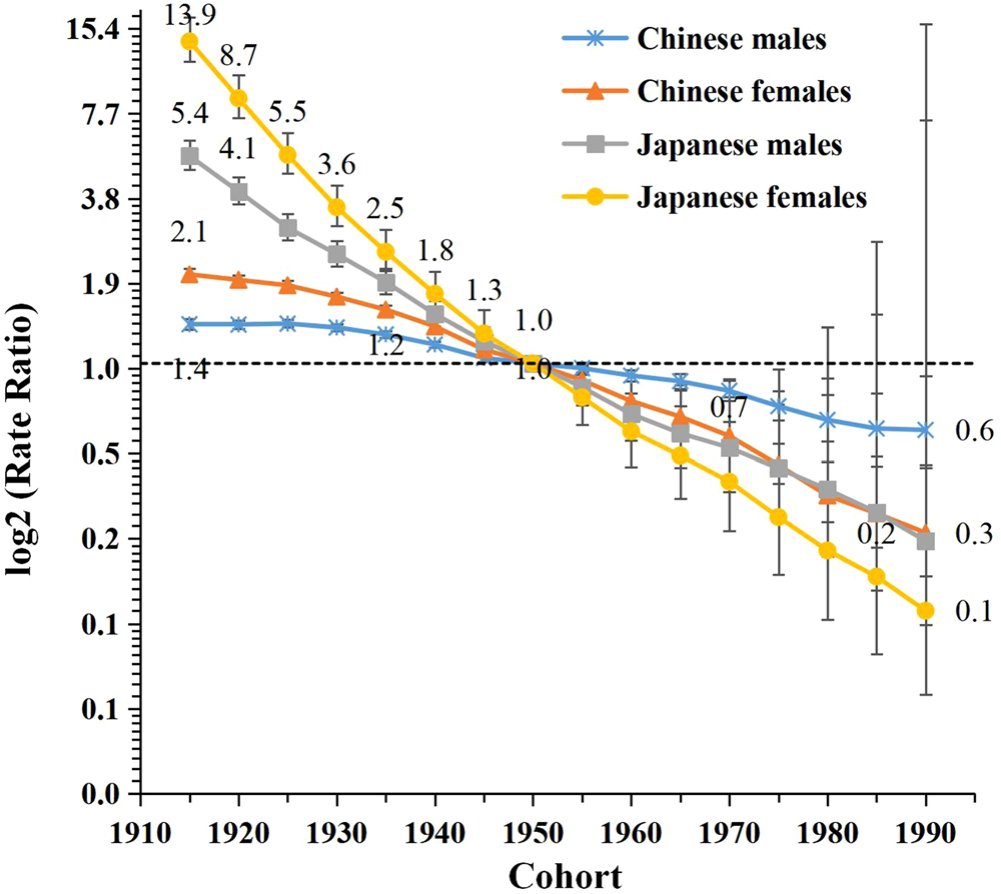
Cohort relative risks (RRs) of LPA-attributable stroke mortality rate by sex in China and Japan. The relative risk of each cohort compared with the reference one (cohort 1950) adjusted for age and nonlinear period effects. Error bars represent the 95%CIs for the cohort relative risks.

The estimated sex-specific period and cohort RRs in China and Japan are depicted in Figs. 5 and 6. The period RRs showed decreasing trends for both sexes in both countries, with a faster decline for women compared to men and for Japanese compared to Chinese. Although Chinese have experienced lower LPA-attributable stroke mortality than Japanese during the 1990s, RRs appeared to cross in the year 2000, then decreased slower among Chinese compared to Japanese. Similarly, the Japanese and Chinese cohort RRs crossed in 1950, with higher LPA-attributable stroke mortality rates before then among Japanese, but lower rates afterward compared to Chinese. The cohort RRs showed decreasing patterns for both sexes in both countries with a more substantial decline for women than for men in all birth cohorts, especially in Japan where the RR for women decreased from 13.9 to 0.1. The cohort and period RRs were statistically significant for both sexes in both countries (p < 0.01), as well as the net and local drifts with the exception of the local drift for Japanese men (Table 1).

**Table 1:** Wald Chi-Square tests for estimable functions in the APC model.

Null Hypothesis	China				Japan			
	Males		Females		Males		Females	
	Chi-Square	*P-*Value	Chi-Square	*P*-Value	Chi-Square	*P*-Value	Chi-Square	*P*-Value
NetDrift = 0	155.75	<0.001	469.07	<0.001	29.40	<0.001	55.71	<0.001
All Period RR = 1	198.80	<0.001	819.20	<0.001	53.17	<0.001	70.12	<0.001
All Cohort RR = 1	1033.18	<0.001	2999.40	<0.001	1770.45	<0.001	2049.69	<0.001
All Local Drifts = Net Drift	103.24	<0.001	143.14	<0.001	9.09	0.613	28.03	0.003

for the main figures.

## Discussion

During the period between 1990 and 2016, clear downward trends of ASMRs and cohort/period RRs were observed for LPA-attributable stroke among Chinese and Japanese. In both countries, the net and local drift curves were below zero in all age groups for both sexes. However, the local drift for Japanese decreased with each increasing age group in contrary to that for Chinese.

For decades, a declining trend for stroke mortality was observed in Japan^27^, and overall in China as well^2,28^. These trends were explained partly by stroke care improvement and risk factor management through the promotion of a healthier lifestyle including decreased salt and alcohol intake, smoking cessation, and physical activity^29,30^. LPA was associated with an increased risk of stroke mortality in both countries^31–33^. The reasons for the increased mortality among physically inactive adults include a generally worse health status due to the high prevalence of hypertension, type 2 diabetes, and obesity; all of which in itself might constitute to increased stroke risk^34,35^.

In China, the proportion of the physically active population increased from 6.3% in 1989^36^ to 11.9% in 2010^37^, while among Japanese men and women, respectively, these proportions were 21.9% and 18.1% in 1990, and 34.8% and 28.5% in 2010^38,39^. However, according to the China Health and Nutrition Survey, the proportion of PA Chinese did not significantly change between 1997 (12.97%) and 2000 (11.55%), which might partly explain the steady ASMRs for LPA-attributable stroke during the corresponding period (1995–2000)^36^. In this study, people aged 60 years or older had >90% of stroke mortality attributable to LPA in both China and Japan. However, local drift curves showed a larger yearly decline in LPA-attributable stroke mortality among older than among younger Japanese, while in China the opposite was observed. This result might be partly explained by the differences in the distribution of the physically active population within the two countries. The Japanese National Health and Nutrition Examination Survey in 2010 reported that the proportion of physically active Japanese was 18.7% among those aged 20–29 years and 61.4% among those aged over 65 years^39^. Contrarily, data from the Chinese Chronic Disease Surveillance 2010 indicated 15.6% physically active Chinese for the age group 18–24 years and 9.9% for those aged 75 years and over^37^. Moreover, the Chinese Epidemiological Survey conducted in 2010 showed that 85.4% of older Chinese (aged ≥ 60 years) did not engage in leisure-time PA, and only 12% were physically active^40^.

The results also showed that the period and cohort RRs of LPA-attributable stroke mortality in Japan and China crossed in the years 2000 and the cohort born in 1950, respectively, with higher RRs in Japan before then but higher RRs in China afterward. In general, the Japanese population was considered to be at a higher risk for stroke morbidity and mortality than the Chinese due to higher smoking prevalence among men (53.1% in Japan^39^ versus 35% in China^41^), higher salt intake (25–30 g/d in Japan^42^ versus 15 g/d in China^43^), and higher mean systolic blood pressure (131.6 mmHg in Japan^39^ versus 124 mmHg in China^44^). However, after 2000 many strategies have been implemented in Japan to counter the high stroke mortality rates which lead to a rapid decline in smoking prevalence, salt intake, and blood pressure levels^39^. In 2004, the Japan Stroke Society, the Japanese Society of Neurology, the Japan Neurosurgical Society, the Japanese Society of Neurological Therapeutics, and the Japanese Association of Rehabilitation Medicine formulated jointly the Japanese Guidelines for the Management of Stroke which was completed in its first revision in 2008. The guidelines recommended that insufficient physical activity should be avoided as primary and secondary preventive measures^45^. Accordingly, the proportion of physically active Japanese increased from 24.1% in 1995 to 31.7% in 2015^39^. On the other hand, the implementation of related PA policies for stroke prevention in China was delayed. The Chinese Cerebrovascular Disease Prevention and Control Guide in 2013 recommended engaging in PA at least 3 to 4 days/week, 40 min/time (walking fast, jogging, riding, or other aerobic exercises) for stroke prevention^46^. However, with China’s economic growth during the past three decades, access to public spaces for sports or leisure activities was reduced (e.g., parks, sports areas, and open recreational fields) due to increased urbanization and motorization (from 12 vehicles per 1000 people in 2002 to 82 in 2012)^46^.

In recent decades, the mean sex ratio of stroke mortality (men to women) experienced an increasing trend worldwide. Stroke mortality generally decreased during the same period in both sexes. However, women experienced lower mortality rates and a faster decline than men, similarly to the findings of this study^47^. In Japan, the mean sex ratio of stroke mortality increased from 1.46 in the period 1954–1956 to 2.00 in 1986–1991 among the population aged 55–64 years^47^. The reasons for the sex difference in stroke mortality are still not clear, but may partly be attributed to the different extent of lifestyle interventions in women compared to men. Previous findings in the published literature also suggested that PA might have a greater effect on women’s cerebrovascular health compared to men’s^31,34^.

From the beginning of the century, the period and cohort effects showed declining trends for stroke mortality in China and Japan^2,48^ which could be partly attributed to antihypertensive treatment, socioeconomic status improvements, and lifestyle changes including increased PA level^49^. However, previously there were no data on LPA-attributable stroke mortality trends in these countries or worldwide. The results of this study showed that period effects of the LPA-attributable stroke mortality decreased in both sexes in both countries throughout the study period. However, currently the burden of stroke is still great in Asia, and stroke mortality is also higher than in Europe and North America^29^. Physically inactive individuals still constitute approximately half of East Asians and 24% of US citizens^50,51^. One of the reasons for this may be that the recommended PA standards are too high to initiate or continue. For example, the standard of the World Health Organization is 150 min/week of moderate-intensity exercise or 75 min/week of vigorous-intensity exercise^52^. The current stroke guidelines of the American Stroke Association recommended at least 40 min/day of moderate- to vigorous-intensity aerobic exercise 3 to 4 days/week^53^. According to these standards, 80% of all adults failed to meet the standards in Asian countries such as China, Japan, or Taiwan^51,54,55^. On the other hand, in Korea the introduced standards for PA include vigorous-intensity exercise for ≥10 min/time, ≥20 min/day, and ≥3 days/ week, or moderate-intensity exercise/walk for ≥10 min/time, ≥30 min/day, and ≥5 days/week^56^. Data from the Korea National Health and Nutrition Examination Survey 2010 reported that nearly 50% of the population aged ≥19 years met the recommended PA level, and the proportion of physically inactive people decreased from 68.5% to 50.8% from 2005 through 2010^57^. Based on this outcome, since 2015 as the new recommendation the Japanese government is encouraging people to practice a minimal starting dose of +10 min/day of moderate to vigorous exercise, urging them to become more active progressively^58^.

This is the first study to analyze the effects of age-period-cohort on and the temporal trends of LPA-attributable stroke mortality, focusing on the comprehensive comparison of China and Japan. Data of the GBD 2016 has provided internally consistent estimates of age- and sex-specific all-cause and cause-specific mortality, which had sufficient quality to reduce the possibility of misclassification of outcomes. However, there are several limitations to this study. First, similarly to other APC analyses, the possibility of ecological fallacy cannot be excluded; interpretation of results at population level does not necessarily hold at the individual level. Therefore, the findings of this work need to be confirmed in the future using individual-based studies. Second, people aged 80 years or older could not be analyzed in this study since they were recorded as one group in the GBD database while period and age intervals should be fixed and equal in the APC tool. However, previous studies confirmed that the stroke mortality rate of those aged ≥80 years is decreasing as well, showing a similar pattern to other age groups^2,25^. Third, data on individuals aged 0–25 years were excluded because of the negligible LPA-attributable stroke mortality rate in this group. Fourth, due to the lack of detailed Japanese data, stroke subtypes such as ischemic stroke and hemorrhagic stroke could not be analyzed.

In summary, the ASMRs and the period and cohort effects of LPA-attributable stroke mortality in China and Japan were declining in both sexes and all age groups between 1990 and 2016. However, the proportion of the physically active population is still small in both countries. Consequently, policymakers should concentrate on further promoting PA, which should be one of the most recommended effective strategies in stroke prevention, especially in China.

## Supplementary information

Supplementary Table 1.
