# Supplementary material for: An Age-Period-Cohort Analysis of Stroke Mortality Attributable to Low Physical Activity in China and Japan: Data from the GBD Study 1990–2016
Source: Sci Rep. 2020 Apr 16;10:6525. doi: 10.1038/s41598-020-63307-x (PMC7162896; doi:10.1038/s41598-020-63307-x)
Supplement: Supplementary file 1 — Supplementary Table 1. [file 41598_2020_63307_MOESM1_ESM.pdf]

Supplementary Table 1 The number of population and stroke in Japan and China(1990-2016).

Year	Japanese men		Japanese women		Chinese men		Chinese women	
	Population	Stroke	Population	Stroke	Population	Stroke	Population	Stroke
1990	38,067,229	38,690	40,384,673	38,284	290,646,432	2,169,970	275,594,048	1,336,893
1995	40,061,997	43,172	42,106,114	36,083	337,919,965	2,769,952	322,776,513	1,643,057
2000	42,325,105	53,896	44,166,128	33,924	380,94,008	3,551,441	365,152,409	2,082,019
2005	43,537,328	56,710	45,113,407	27,271	409,095,096	4,620,059	393,674,755	2,545,186
2010	43,9242,91	51,523	45,222,792	21,438	436,732,774	5,592,961	421,787,480	2,723,681
2016	43,374,707	49,693	44,430,983	19,873	483,290,492	7,389,853	466,392,696	3,082,721
